# Comprehensive 4D Parallel Transmission Spatial‐Spectral Pulse Design for Slab‐Selective Uniform Water‐Selective Excitation: Demonstration in the Human Brain at 7 Tesla

**DOI:** 10.1002/mrm.70183

**Published:** 2025-11-14

**Authors:** Xin Shao, Zhe Zhang, Wen Zhong, Hua Guo, Kamil Ugurbil, Xiaoping Wu

**Affiliations:** ^1^ Center for Biomedical Imaging Research, School of Biomedical Engineering Tsinghua University Beijing China; ^2^ Tiantan Neuroimaging Center of Excellence, Beijing Tiantan Hospital Capital Medical University Beijing China; ^3^ Center for Magnetic Resonance Research, Radiology, Medical School University of Minnesota Twin Cities Minneapolis Minnesota USA

**Keywords:** parallel transmission, radiofrequency pulse design, slab‐selective excitation, spatial‐spectral pulse design, water‐selective excitation

## Abstract

**Purpose:**

To propose a new parallel transmission (pTx) spatial‐spectral (SPSP) pulse design for achieving slab‐selective uniform water‐selective excitation without unwanted out‐of‐slab fat excitation when using bipolar slab‐selective gradients to maintain a sharp slab profile.

**Methods:**

Our new pTx SPSP pulses were designed by formulating the design problem comprehensively in the 4D space (1D spectral and 3D spatial domains) and by incorporating a SPINS‐like 2D excitation k‐space trajectory for within‐slab flip‐angle homogenization. Our new design was validated at 7 T using simulation, phantom and human experiments with the commercial Nova eight‐channel transmit RF head coil. Its utility was demonstrated by comparing to traditional multi‐spoke pTx SPSP pulses.

**Results:**

In both simulation and experiments, our design outperformed traditional approaches, producing slab‐selective uniform water‐selective excitation with no out‐of‐slab fat excitation. Quantitatively, coefficient of variation measuring excitation non‐uniformity reduced by up to ∼23%.

**Conclusion:**

Our proposed new design provides an effective solution for slab‐selective uniform water‐selective excitation, holding a promise to many applications including mesoscale BOLD fMRI and fat‐free body imaging at ultrahigh field.

## 1 Introduction

Radiofrequency (RF) parallel transmission (pTx) [1, 2, 3, 4] is a leading‐edge technology that allows channel‐wise RF waveforms to be applied through a multi‐channel RF coil. It offers an effective solution to two significant challenges often encountered in ultrahigh‐field (UHF) MRI: transmit B1 (B1+) inhomogeneity and specific absorption rate (SAR) [5, 6]. Its efficacy for counteracting B1+ inhomogeneity and improving flip angle uniformity while reducing SAR has been demonstrated for many UHF MRI applications in the human body [7, 8] and brain [9, 10, 11, 12].

In many MRI applications, receiving only water signal at the echo time is crucial for eliminating chemical‐shift artifacts caused by fat signal contamination. An effective way to achieve this is by water‐selective excitation (water‐excitation), in which only water magnetization experiences a net nutation during the excitation process. Water‐excitation has advantages over other fat‐suppression approaches, such as reduced SAR relative to those based on fat saturation [13, 14, 15, 16] and increased signal‐to‐noise ratio (SNR) relative to those based on inversion recovery [17, 18].

One practical way to fulfill water‐excitation is by spatial‐spectral (SPSP) RF pulse design [19]. In addition to their 1D spectral selectivity, SPSP pulses can be designed to have 2D [20, 21] or 3D [22, 23] spatial selectivity, depending on their imaging application. Previous studies have demonstrated how pTx SPSP pulses can be designed for water‐excitation using spoke [24], kT‐point [25], and SPINS [26] trajectories. Most recently, we have proposed a new method for designing pTx SPSP pulses and demonstrated how it can be used with kT‐point [27] and SPINS [28] parameterizations to design pulses to achieve uniform water‐excitation across the entire brain at 7 T [29]. This new method is believed to have a utility for any volumetric imaging applications at UHF that will benefit from fat suppression while allowing spatially non‐selective RF excitation. However, for many other imaging applications that require exciting a single image slab (e.g., UHF layer‐specific functional MRI [30] usually performed with partial brain coverage), it remains unclear how best to design pTx SPSP pulses that can produce slab‐selective uniform water‐excitation.

Here, we propose a new 4D pTx SPSP pulse design and demonstrate its utility for slab‐selective uniform water‐excitation at 7 T, some of this work was previously presented in abstract form at the ISMRM Annual Meeting [31]. In our new design, the pulse design problem is formulated by prescribing a 4D excitation target and considering different regions in a hybrid 1D spectral and comprehensive‐3D spatial domain. This is to eliminate fat “odd lobes” [32] while improving slab profiles when using bipolar slab‐selective gradients. We examined the effectiveness of our new design using the commercial Nova (Wilmington, MA, USA) 8‐channel transmit 32‐channel receive (8Tx32Rx) head RF coil. The efficacy of our new design for slab‐selective uniform water‐excitation was first evaluated via simulation. Our new design was then validated in both phantom and human experiments using a custom pTx‐enabled gradient‐echo (GRE) sequence. Our results showed that our new design outperformed traditional pTx SPSP multi‐spoke pulse design approaches in producing robust uniform water‐excitation in the imaging slab. As such, our new design has potential to facilitate many UHF applications including partial‐brain mesoscale functional MRI and fat‐free body imaging at 7 T and above.

## 2 Methods

All pTx pulses were calculated in Matlab (The Mathworks Inc., Natick, MA, USA) and on a PC (Intel 10900K with 10 cores, 128 GB RAM). The source code for our pulse design is downloadable at https://github.com/longint1024/Real‐4D‐pTx. Phantom and human experiments were performed on a Siemens Magnetom Terra (Siemens, Erlangen, Germany), equipped with whole‐body gradient coils (capable of 200 T/m/s maximum slew rate, and 70 mT/m maximum amplitude) and capable of 8‐channel pTx and up to 64‐channel receive operation. All data were acquired using the commercial Nova 8Tx32Rx head coil (Nova Medical Inc., MA, USA). For proof of principle, one healthy adult was scanned by designing our proposed 4D pTx SPSP pulses who signed a written consent form approved by the local Institutional Review Board at Tsinghua University prior to scan.

### 2.1 Pulse Design

**FIGURE 1:**
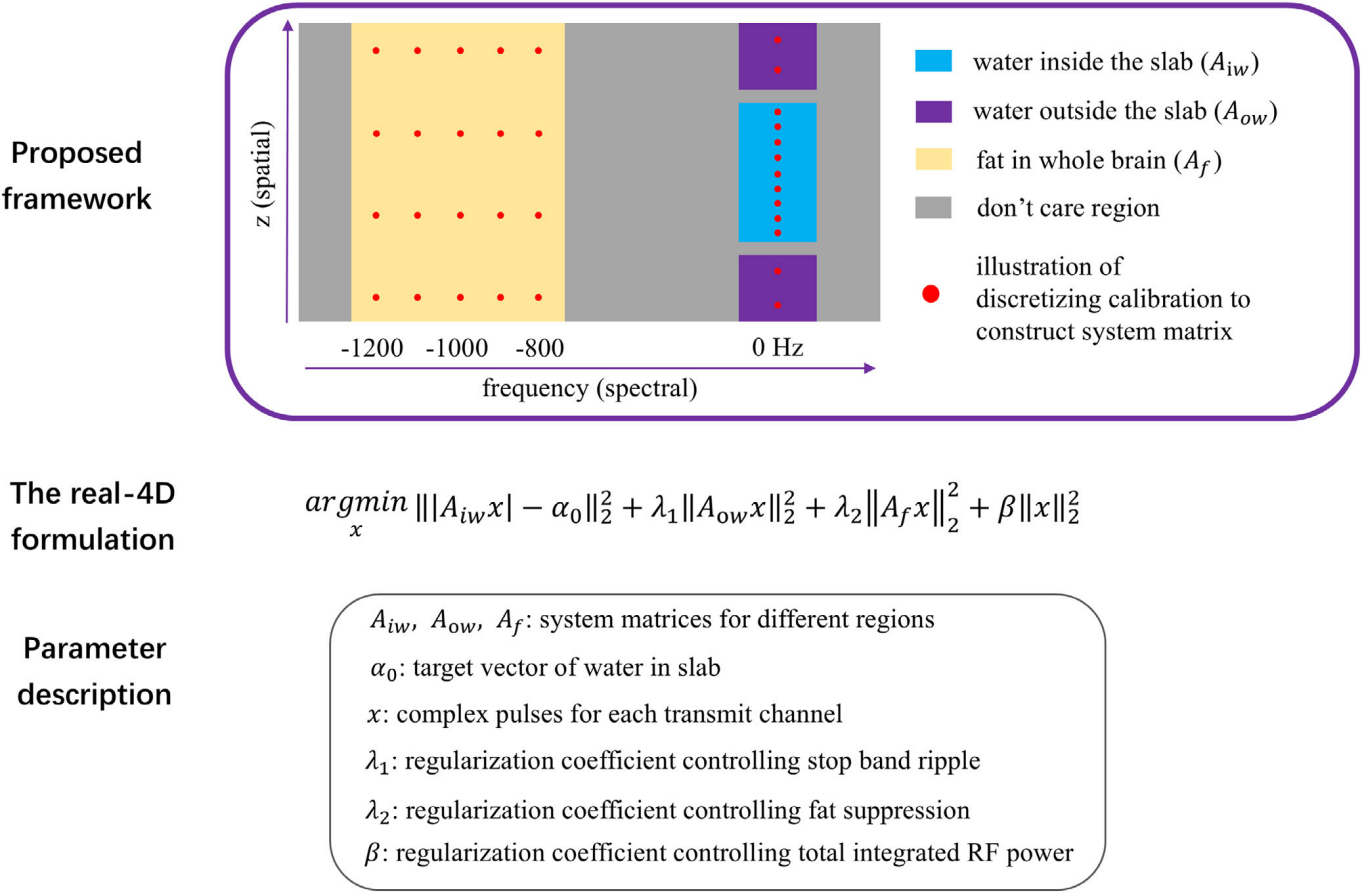
Framework for our proposed comprehensive‐4D parallel transmission (pTx) spatial‐spectral (SPSP) pulse design for slab‐selective uniform water excitation. Design problem is formulated by prescribing a comprehensive 4D excitation target (i.e., in the hybrid 1D spectral and comprehensive 3D spatial domains) and considering four regions (top row): Water inside the slab, water outside the slab, fat, and don't care region, with red dots illustrating how calibration can be sampled to construct system matrices for the formation of the design problem. For proof of principle and without loss of generality, the design problem used here (middle row) was formulated in the small tip angle regime to be regularized magnitude least squares optimization, where theta represents prescribed parameters determining the 3D excitation k‐space trajectory, lambda and beta the regularization parameters used to balance the tradeoff between excitation fidelity (as dictated by the first term), stopband suppression (the middle two terms), and total RF power (as dictated by the last term).

With our new pulse design (Figure 1), the design problem was formulated by prescribing a comprehensive‐4D excitation target (i.e., in 1D spectral and comprehensive 3D spatial domains) and considering four regions: inside‐slab water, out‐of‐slab water, fat, and don't‐care region (i.e., not considered in pulse design). The spectral and spatial components of the 4D excitation target were prescribed to dictate uniform 3D in‐slab excitation with no out‐of‐slab excitation at the water resonance (i.e., 0 Hz), together with a 400‐Hz wide fat stopband defined on five frequency points surrounding the fat resonance (i.e., −1200, −1100, −1000, −900, and −800 Hz) but spanning the entire brain in the 3D space. Mathematically, the design problem was formulated in the spatial domain [33] as regularized magnitude least squares (MLS) [34] assuming small tip angles:

(1) $$\underset{x}{\text{argmin}}{\left\|\left|A_{\mathrm{iw}}x\right|-\alpha_{0}\right\|}_{2}^{2}+\lambda_{1}{\left\|A_{\mathrm{ow}}x\right\|}_{2}^{2}+\lambda_{2}{\left\|A_{f}x\right\|}_{2}^{2}+\beta {\left\|x\right\|}_{2}^{2}$$

Here *x* denotes a vector concatenating RF waveforms of individual transmit channels; *A*_iw_, *A*_ow_, and *A*_f_ denote the system matrices for inside‐slab water, out‐of‐slab water, and fat, respectively; $\alpha_{0}$ is a scalar representing the target flip angle for inside‐slab water; $\lambda_{1}$ and $\lambda_{2}$ are scalars used to control the balance between suppression and excitation; β is the regularization parameter that can be adjusted to control the tradeoff between excitation error (evaluated by the first three terms) and total integrated RF power (dictated by the last term). We note that the formulation in Equation (1) implies that the target flip angle is 0° for both fat and out‐of‐slab water. The design problem in Equation (1) was solved using the Variable Exchange Method (VEM) [34, 35], with $\lambda_{1}$ and $\lambda_{2}$ set to 2 and 8, respectively. The parameter β was set to 2000 unless noted otherwise. A description of how $\lambda_{1}$, $\lambda_{2}$, and β were chosen is provided in the Supporting Information. The target flip angle $\alpha_{0}$ was set to ∼13° (i.e., the Ernst angle assuming a TR of 50 ms and a gray matter $T_{1}$ of ∼2 s at 7 T [36]).

Calibration data needed for pTx pulse design was obtained using a custom workflow [29]. Briefly, subject‐specific whole brain ∆*B*_0_ mapping, multi‐channel B1+ mapping [37, 38, 39, 40, 41], and brain masking [42] were acquired using vendor product sequences. The ∆*B*_0_ mapping was performed with a double‐echo multi‐slice 2D GRE sequence and the multi‐channel B1+ mapping with the vendor‐provided mapping tool based on a tailored multi‐slice 2D sequence. DICOM images (output by the associated product sequence) were exported and postprocessed using a custom Matlab routine, resulting in B1+ and ∆*B*_0_ maps in 80 axial slices at 3‐mm isotropic resolutions. Before calibration, second‐order *B*_0_ shimming was performed to improve static field homogeneity across the ROI and was kept unchanged throughout the scan session.

For improved computation efficiency, calibration data (including ∆*B*_0_ and B1+ maps) were down‐sampled in space to construct the three system matrices (i.e., *A*_iw_, *A*_ow_, and *A*_f_) in the design problem in Equation (1). To maximally keep the design performances, a region‐specific down‐sampling strategy was employed in‐plane and through‐plane for the three associated regions. For the region of inside‐slab water, calibration data were only down‐sampled in‐plane to 6‐mm resolution to construct matrix *A*_iw_. For both regions of fat and out‐of‐slab water, calibration data were down‐sampled in‐plane to 18‐mm resolution as well as through‐plane to 9‐mm resolution to construct matrices *A*_ow_, and *A*_f_. The discretization time step was set to 2μs. The computation time was 17 min 53 s unless noted otherwise.

The target slab profile was defined by including a transition band on either side of the passband (corresponding to the inside‐slab water region). Both transition bands were considered part of the “don't‐care” region. Furthermore, the width of either transition band was prescribed to be 10% of that of the passband. The effective slab thickness (measured as the FWHM of the slab profile) was thus given by 1.1 times the thickness of the inside‐slab water region (associated with the width of the passband). The thickness of the inside‐slab water region was set to 48 mm corresponding to an effective slab thickness of 52.8 mm unless noted otherwise.

Our pTx SPSP pulses were designed using prescribed 3D gradient waveforms that consisted of 1D slab‐selective and 2D in‐plane gradient waveforms. The 1D slab selective gradient waveform was designed to have three pairs of bipolar trapezoids (oscillating at a period of 1 ms) followed by a rewinder, leading to a total pulse duration of ∼3.2 ms. The in‐plane gradient waveforms of the same length were designed to have a 2D k‐space trajectory similar to SPINS [28] and were determined by $k\left(t\right)=\left(r_{k}\left(t\right)\sin\theta_{k}\left(t\right)\cos\varphi_{k}\left(t\right),r_{k}\left(t\right)\sin\theta_{k}\left(t\right)\sin\varphi_{k}\left(t\right),r_{k}\left(t\right)\cos\theta_{k}\left(t\right)\right)$ where rk(t)=kmax1+expαtTp−β, $\theta_{k}\left(t\right)=\mathrm{ut}$, $\varphi_{k}\left(t\right)=\mathrm{vt}$ with parameters set to values same as the initial values in our previous study [29], that is, $k_{\max}=20$ rad/m, α=10, β=0.5, u=8π rad/ms, and v=2π rad/ms.

### 2.2 Simulation Study

**FIGURE 2:**
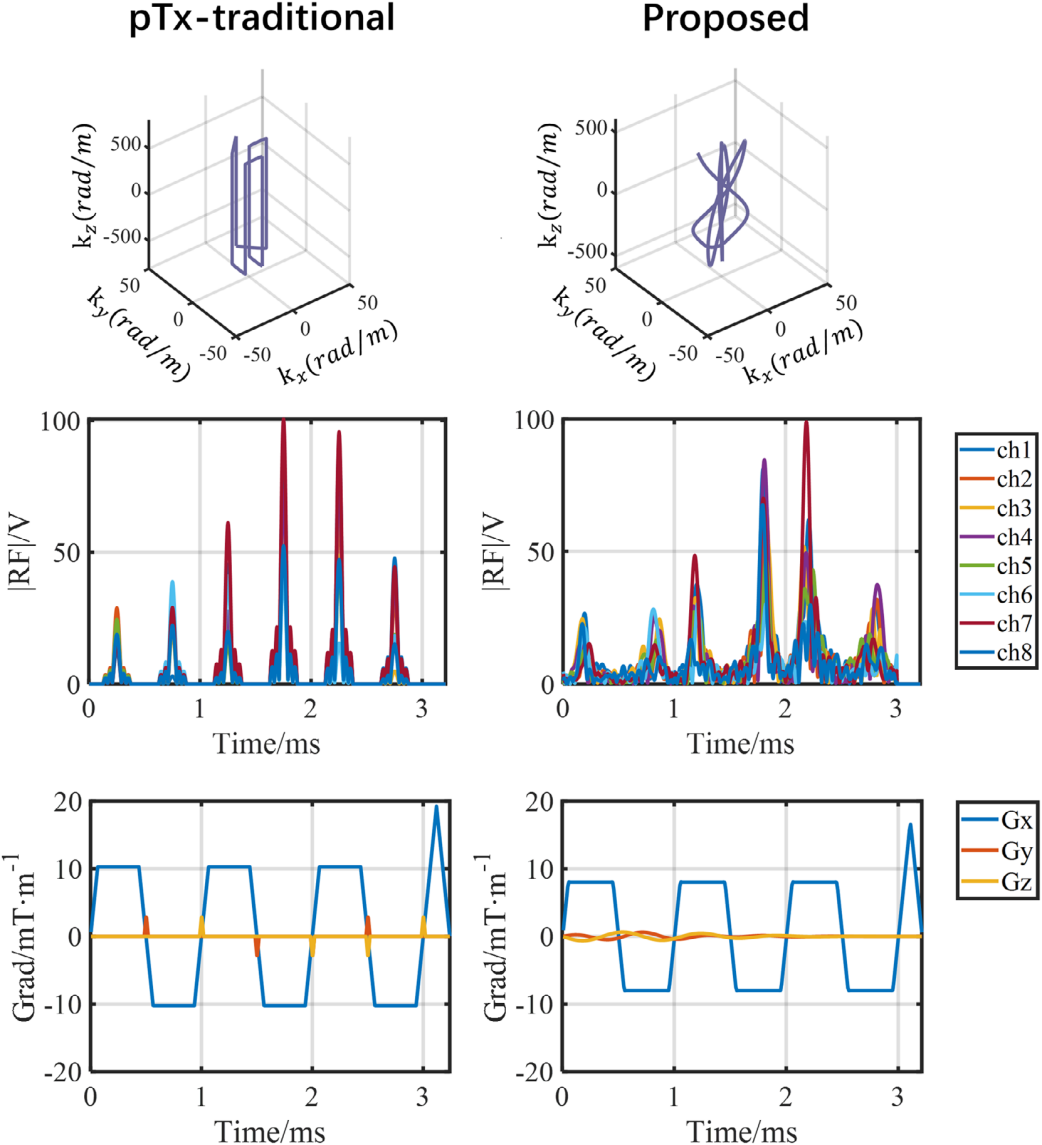
Example pulse design (proposed) in comparison to traditional pTx SPSP multi‐spoke pulse design (pTx‐traditional). For each, displayed are k‐space trajectories (top), RF amplitudes (middle), and corresponding gradient waveforms (bottom). Both pTx pulse designs were performed using pTx calibration (i.e., ∆*B*_0_ and multichannel transmit B1 maps) obtained in humans at 7 T with the commercial Nova 8‐channel transmit 32‐channel receive (8Tx32Rx) RF head coil. All pulses were designed to have a pulse duration of ∼3.2 ms.

Simulation was conducted to compare our pulse design to existing approaches. Calibration data (including B1+ mapping, $∆B_{0}$ mapping and brain masking) obtained from a single healthy volunteer at 7 T using the Nova 8Tx32Rx coil was used for our proposed pTx pulse design. Our pTx pulses (Figure 2) were designed to excite a coronal slab at the gradient isocenter. The performances of our pTx pulses were then evaluated based on Bloch simulations, which were run to create flip angles within all 80 calibration slices encompassing the entire brain. For both water and fat resonances, the slab profile was also measured by tracing respective flip angle variations in the slice direction along the centerline within the central sagittal slice.

For evaluating the quality of slab‐selective uniform water‐excitation, root mean square error (RMSE) with respect to the target (i.e., 0° for fat and out‐of‐slab water, and 13° for inside‐slab water) was computed. Coefficient of variation (CoV) of water‐excitation inside the slab was also calculated to quantify the uniformity of the slab‐selective water‐excitation.

For comparison, traditional pTx SPSP pulses (pTx‐traditional) of the same duration were designed using spoke parameterization [8, 11, 43, 44]. The pTx‐traditional pulses were designed using the same calibration data as in our comprehensive‐4D pTx SPSP pulse design, for which 3 calibration slices spanning the slab of interest were under‐sampled to 6 mm in‐plane resolution to form the design problem. The design problem was formulated as regularized MLS to calculate channel‐ and spoke‐wise RF weights for achieving uniform water‐excitation inside the slab. In‐plane gradient blips were prescribed to place six spokes including five near and one at the center of the excitation k‐space. The six spokes meant for slab selectivity corresponded to three pairs of bipolar slab‐selective trapezoidal gradients (similar to those used in our comprehensive‐4D pulse design). The sub‐pulse used to form the final composite pTx pulses was a sinc‐shaped pulse of ∼0.3 ms in length and with a time bandwidth product (TBWP) of 6.

We also studied the sensitivity of our proposed 4D pTx SPSP pulses to potential gradient delays. This was done by introducing a gradient delay relative to the RF pulses and evaluating the associated pulse performances with Bloch simulation. The gradient delay considered here ranged from −5 to +5 μs in steps of 1 μs. In each case, the same gradient delay was applied to all gradient axes, assuming isotropic delays.

To investigate how robust our proposed 4D SPSP pulse design would be against anatomic variability, we designed pulses for nine additional volunteers. This was done by designing subject‐specific pulses as described above using experimentally acquired calibration data at 7 T using the same Nova RF coil. For each volunteer, pulse performances were evaluated with Bloch simulation.

### 2.3 Experimental Validations

We validated our proposed pTx SPSP pulse design method by performing both phantom and human experiments. For both, calibration data were obtained using the same custom workflow as in our simulation study. Slab‐selective pTx water‐excitation pulses were designed to excite a coronal slab. The efficacy of our designed pulses was examined by applying them in a custom pTx‐enabled GRE sequence [29]. Online local SAR supervision provided by the vendor was fulfilled using the commercial virtual observation points [45] model of the Nova coil.

In phantom experiments, we scanned a spherical phantom of 16 cm in diameter and filled with doped water (obtained by adding NiCl_2_ × 6H_2_O [1.32 g per 1000 g H_2_O] and NaCl [8 g per 1000 g H_2_O] to distilled water to reduce T1 and increase conductivity values). The frequency response of our pulses was measured by acquiring a series of 3D GRE images as a function of RF frequency offset. This was done by incrementing the frequency offset from −1300 to 300 Hz in steps of 100 Hz and acquiring the corresponding 3D GRE image. GRE images were acquired at isotropic 3‐mm resolution, other relevant imaging parameters being FOV = 180 × 180 × 180 mm^3^, orient = coronal, TE/TR = 8/50 ms, nominal FA = 13°, in‐plane GRAPPA factor (iPAT) = 2, and total acquisition time = 1 min 53 s. The series of 3D GRE images were further used to derive the SPSP response pattern of our pulses characterizing image intensities as a function of frequency offset and *z* location (i.e., the location in the slice direction). This was done by (1) interpolating 3D GRE images in the frequency offset dimension (to have a denser image intensity definition in steps of 10 Hz), and (2) averaging image intensities at each frequency offset across three centerlines traversing in the slice direction in the middle axial slice. For comparison, the SPSP response pattern of our pulses was also created using Bloch simulation performed at 3‐mm isotropic resolution in space and at 10‐Hz resolution in frequency.

In human scans, we collected 3D GRE brain images at higher resolution to demonstrate the utility of our proposed pulse design. In particular, slab selective pTx water‐excitation pulses were designed to excite a coronal slab of 52.8 mm in effective slab thickness covering the primary visual cortex. Partial‐brain 3D GRE images were acquired at 0.8‐mm isotropic resolution, other relevant imaging parameters being FOV = 240 × 240 × 52.8 mm^3^, orient = coronal, TE/TR = 8/50 ms, nominal FA = 13°, iPAT = 3, partial Fourier = 6/8 for both phase encoding directions, slice oversampling = 10%, and total acquisition time (including ACS) = 5 min 17 s. For comparison, 3D GRE images were collected in the same volunteer with matched parameters but using (1) pTx‐traditional pulses (designed as in the aforementioned simulation study) and (2) regular RF excitation pulse with the Nova 8Tx32Rx coil operating in its CP mode mimicking single‐channel transmit.

## 3 Results

**FIGURE 3:**
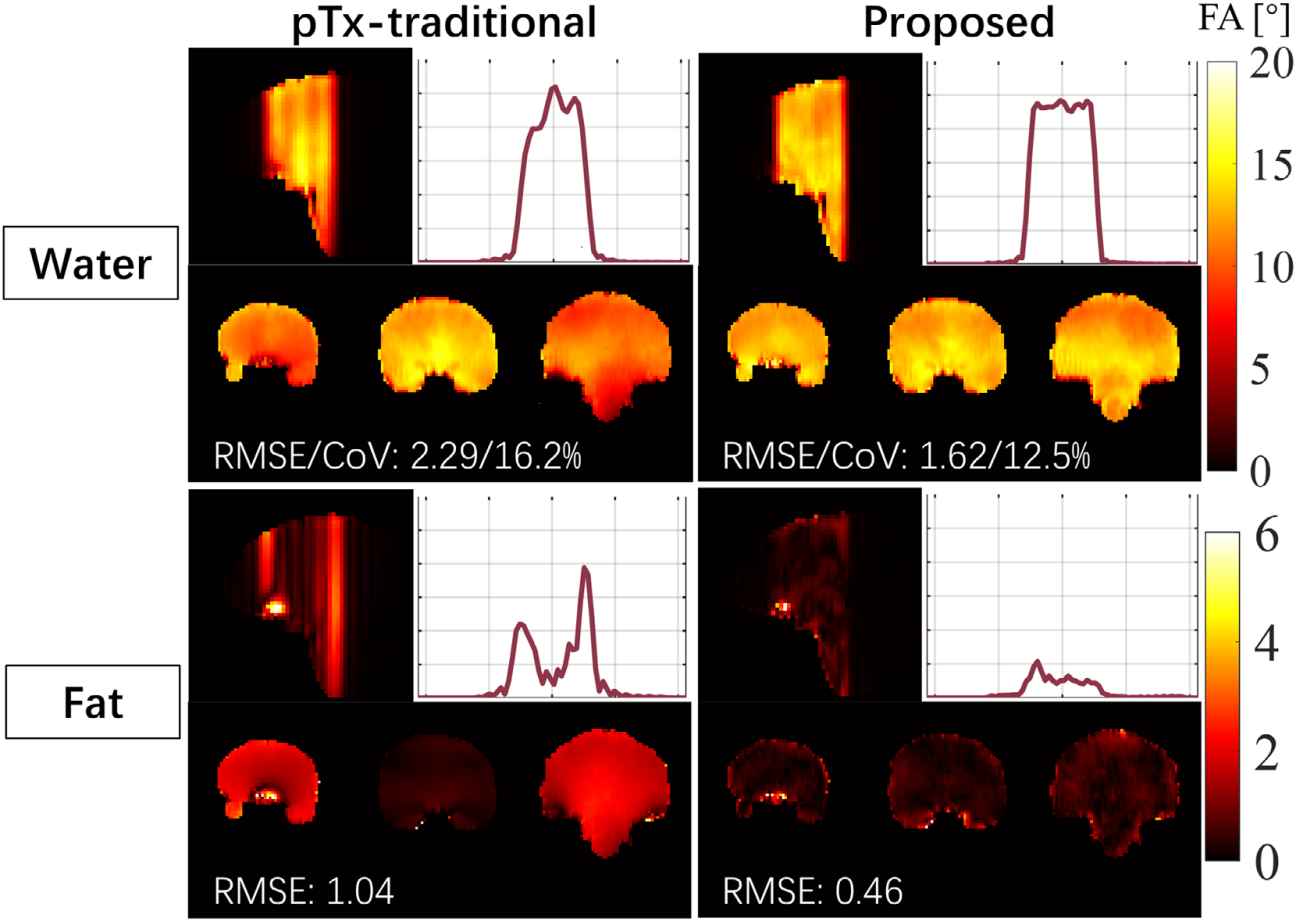
Simulation experiments in a representative volunteer: Evaluating the performances of our proposed pulse design for uniform slab‐selective water‐excitation in comparison to traditional pTx SPSP multi‐spoke pulse design (pTx‐traditional). Shown are Bloch simulated flip angle maps in the middle sagittal slice (alongside corresponding slab profiles) and three representative coronal slices for water (top) and fat (bottom) resonances using pTx calibration obtained in humans at 7 T. Pulses were designed to achieve uniform water excitation within a ∼53‐mm slab in the anterior posterior direction (corresponding to a 48‐mm wide inside‐slab water region along with a 10% transition band on either side). For each pulse design scenario, root mean square errors (RMSE) were computed to evaluate performances for water excitation (calculated with respect to the Ernst angle of ∼13°) and fat suppression (calculated with respect to 0°). Coefficient of variation (CoV) values were also computed to quantify flip angle homogeneity for within‐slab water excitation. Note how our pulse design effectively outperformed traditional pTx SPSP pulse design, leading to uniform slab‐selective water excitation while completely suppressing out‐of‐slab fat.

In simulation, our proposed pulse design achieved quality slab‐selective uniform water‐excitation with a sharp slab profile and without fat odd lobes (Figure 3), outperforming pTx‐traditional pulses. Quantitatively, for water‐excitation, RMSE was reduced by up to ∼29% (1.62 vs. 2.29 for pTx‐traditional) and CoV by up to ∼23% (12.5% vs. 16.2% for pTx‐traditional); for fat suppression, RMSE was reduced by up to ∼56% (0.46 vs. 1.04 for pTx‐traditional).

**FIGURE 4:**
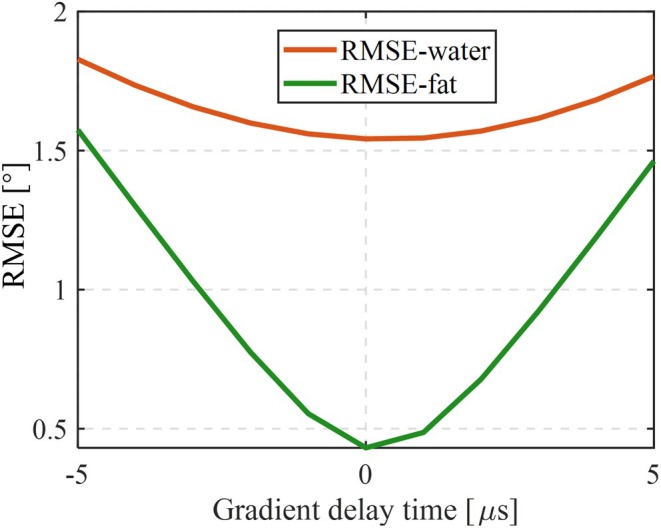
Studying the sensitivity of our proposed 4D pTx SPSP pulses to potential gradient delays. Shown are RMSE values for water excitation (red) and for fat suppression (green) as a function of the gradient delay relative to the RF pulses. The gradient delay ranged from −5 to 5 μs in steps of 1 μs. In each case, the same gradient delay was applied to all gradient axes, assuming isotropic delays. Note how gradient delays affected our pulses' performances especially for fat suppression.

Gradient delays had an effect on our proposed 4D pTx SPSP pulses' performances especially those for fat suppression (Figure 4). Fat suppression performances were found to quickly degrade, with associated RMSE being increased by ∼389% at a ∼5 μs gradient delay. Water excitation performances however were found relatively insensitive to gradient delays, with corresponding RMSE only increased by ∼29% at a ∼5 μs gradient delay.

Our proposed pulse design was found robust against inter‐subject variability (Table 1), performing equally well for all 10 volunteers under consideration. Quantitatively, low RMSE (ranging from ∼1.1° to ∼1.8°) and CoV (ranging from 9% to 14%) values for water‐excitation and low RMSE values (ranging from ∼0.4° to ∼0.6°) for fat suppression were consistently obtained at the subject level. At the group level, the mean RMSE and CoV values averaged across all volunteers were ∼1.57° and ∼12%, respectively, for water‐excitation, whereas the mean RMSE value for fat suppression was ∼0.43°.

**TABLE 1:** Demonstrating the robustness of our proposed 4D pTx SPSP pulse design against inter‐subject variability by designing individualized pulses in 10 volunteers.

Subject index	RMSE‐water (°)	CoV‐water (a.u.)	RMSE‐fat (°)
1	1.81	14%	0.41
2	1.70	13%	0.38
3	1.14	9%	0.55
4	1.72	13%	0.37
5	1.72	13%	0.40
6	1.63	13%	0.46
7	1.56	12%	0.41
8	1.71	13%	0.47
9	1.27	10%	0.42
10	1.43	12%	0.43
Mean ± SD	1.57 ± 0.22	12.20% ± 1.55%	0.43 ± 0.05

*Note*: Shown are subject‐specific RMSE and CoV values for water excitation and associated RMSE values for fat suppression. The bottom row reports the group mean ± standard deviation (SD) across all subjects.

**FIGURE 5:**
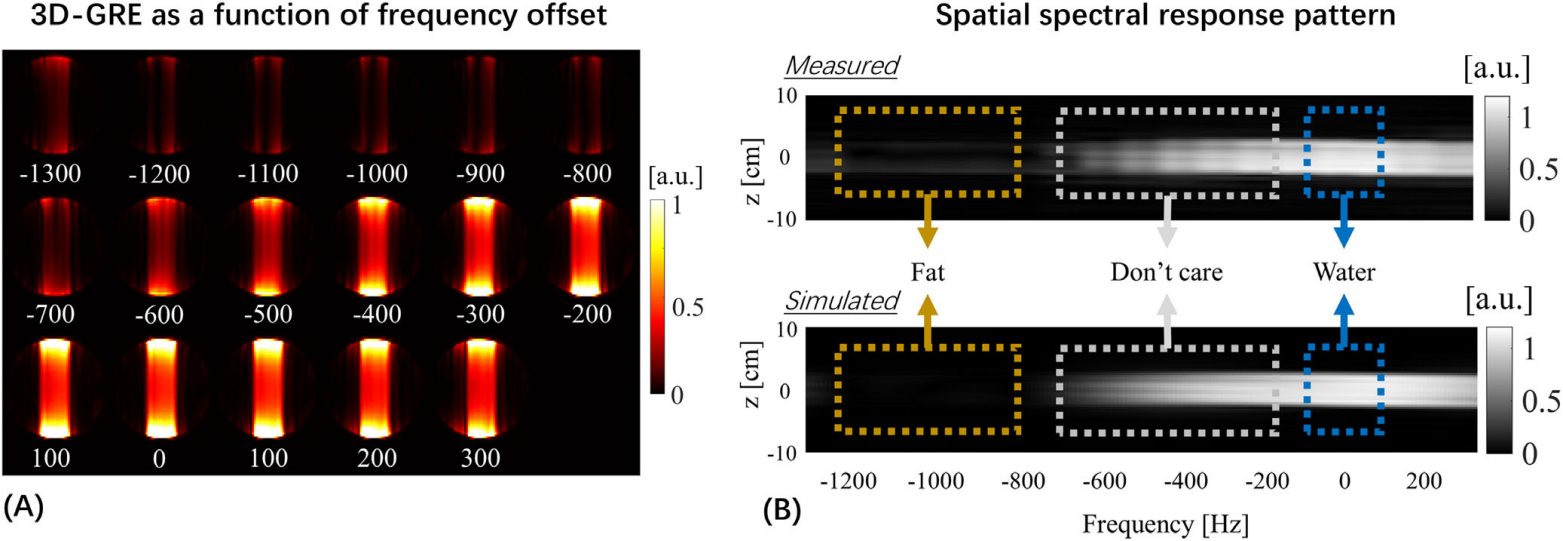
Validating our proposed pulse design in phantom experiments at 7 T using the Nova 8Tx32Rx RF coil. Shown are sagittal views of 3D gradient echo (GRE) images (A) acquired with our pTx SPSP pulses at various frequency offsets ranging from −1300 to 300 Hz in steps of 100 Hz, alongside the associated spatial‐spectral response pattern (B) obtained by interpolating the GRE images in the spectral direction. The spectral component of the excitation target was prescribed to be 1 at the water resonance (i.e., 0 Hz) and 0 at five frequencies around the fat resonance (i.e., ∼−1000 Hz). Note how our pTx SPSP pulses achieved quality uniform slab‐selective excitation around water resonances while suppressing fat across a wide stopband in the hybrid spatial‐spectral domain, producing the desired spatial‐spectral response pattern as predicted by corresponding Bloch simulation.

**FIGURE 6:**
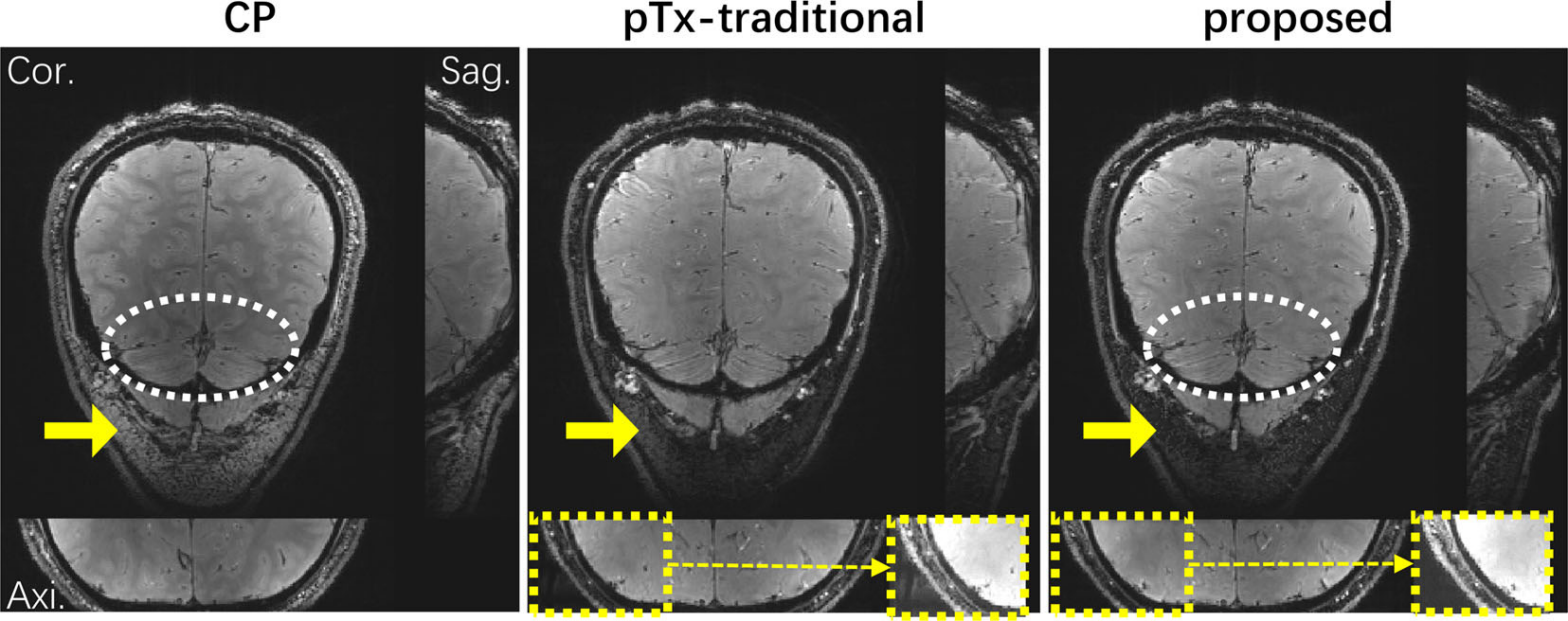
Validating our proposed pulse design in one volunteer at 7 T. Presented are single‐slab 3D GRE images in three orthogonal views acquired using our proposed pTx SPSP pulse design (proposed), in comparison to regular slab‐selective excitation in the CP mode (CP) and traditional pTx SPSP multi‐spoke pulse design (pTx‐traditional). The nominal slab thickness was set to 52.8 mm. All 3D GRE images were acquired at 0.8‐mm isotropic resolution with coronal orientation, 240 × 240 × 52.8 mm^3^ FOV, and 10% slice oversampling. PTx calibration was obtained using a custom workflow based on vendor sequences. Second‐order ∆*B*_0_ shimming inside the image slab was performed. Note that our proposed pulse design resulted in best slab‐selective water excitation, effectively recovering signal in lower brain (as indicated by ovals) while suppressing fat (as indicated by arrows) with chemical shift artifacts eliminated (as indicated by boxes).

With phantom experiments, our proposed pulse design was validated (Figure 5), achieving the desired spatial‐spectral pattern as predicted by Bloch simulations. Likewise, our pulse design was validated in in vivo human experiments and outperformed conventional approaches (Figure 6), improving both water‐excitation (relative to excitation in the CP mode) and fat suppression (compared to pTx‐traditional pulses).

## 4 Discussion

We have introduced a new method for designing 4D pTx SPSP pulses that can achieve quality slab‐selective uniform water‐excitation with bipolar slab‐selective gradients. Our new design method was validated and its advantages over existing approaches demonstrated using simulation, phantom and human experiments. Our results obtained at 7 T using the commercial Nova 8Tx32Rx coil show that our new method can successfully create the desired spatial‐spectral response pattern, outperforming conventional approaches in producing slab‐selective uniform water‐excitation and robust against inter‐subject variability when used for subject‐specific pulse design.

At the heart of our new design is the comprehensive problem formulation by considering four regions in the 4D hybrid domain (1D spectral and 3D spatial) to address out of slab excitation and by introducing a SPINS‐type k‐space trajectory to continuously modulate in‐plane magnetization phases during excitation. This is different than traditional pTx SPSP multi‐spoke pulse design which only considers calibration slices inside the slab of interest and works by assuming that the design problem is separable and can be solved by tackling two sub‐problems: (1) a 4D (1D spectral and 3D spatial) sub‐problem aimed at finding RF weights given 2D in‐plane or 3D gradient blips that can be used to produce uniform water excitation inside the slab of interest, and (2) a 1D subproblem aimed at designing a conventional 1D slab‐selective RF pulse (e.g., sinc) that can be applied during slab‐selective gradients to achieve slab selectivity. This incomplete 4D design can result in undesired fat odd lobes (as shown in Figure 3) when using bipolar slab‐selective gradients.

One way to minimize the undesired fat odd lobes is by using monopolar slab‐selective gradients. However, this will result in a degraded slab profile due in large to having to use sub‐pulses of largely reduced TBWP when keeping the same timing as with bipolar slab‐selective gradients. To demonstrate how the use of monopolar gradients would affect the slab profile, we performed additional simulation by considering slab‐selective water‐excitation. For simplicity and without loss of generality, we assumed single‐channel transmit and opted for binomial pulse design [46, 47], another traditional approach often used for water‐excitation. Specifically, the same binomial coefficients of 1‐5‐10‐10‐5‐1 were used to form two composite RF pulses (Figure S1): (1) one with monopolar and (2) the other with bipolar slab‐selective gradients. Both composite pulses were designed for the same slab thickness and same inter‐pulse duration with their respective optimal TBWP (i.e., the highest possible TBWP). Our calculation (see Supporting Information for detail) showed that using monopolar gradients reduced the optimal TBWP by as much as ∼70% due in large to the addition of fly‐back gradients. The reduced TBWP in turn resulted in a degraded slab profile with worsened sharpness (Figure S2), increasing the non‐uniformity of water‐excitation across the imaging slab. This limitation is expected to remain present when designing traditional pTx SPSP multi‐spoke pulses with monopolar slab‐selective gradients.

Although able to retain the slab profile while effectively eliminating fat odd lobes (Figure 3), our new design appeared to leave residual fat excitation inside the imaging slab despite that a relatively large control parameter (i.e., $\lambda_{2}=8$) had been used to promote fat suppression. Further increasing the control parameter did not seem to completely suppress fat excitation without degrading water excitation. Should the residual fat excitation become a problem in certain applications, it is recommended that our pulses be combined with additional fat saturation (fatsat). To demonstrate the utility of this combination, we performed additional human experiment at 7 T by including vendor‐provided fatsat applied in the CP mode of the Nova 8Tx32Rx coil and compared against a traditional single‐channel transmit approach (i.e., using vendor‐provided regular excitation and fatsat both applied in the CP mode). Our results (Figure S3) showed that our proposed pulse design when coupled with additional fatsat outperformed the traditional single‐channel transmit approach, producing nearly complete fat suppression while creating uniform signal intensity across the imaging slab. Part of our future work is to develop a comprehensive design framework where our new 4D pTx SPSP pulses are designed jointly with our spatially non‐selective pTx fatsat pulses [29] to optimize fat suppression, taking into account hardware and safety constraints. This will likely provide an ultimate pTx solution for slab‐selective uniform water‐excitation, especially when pushing spatiotemporal resolution, for example, in mesoscale functional MRI at UHF using single‐slab 3D GRE EPI. It also remains to be investigated how our proposed 4D pulse design can be extended for high resolution whole brain diffusion MRI when using sequential [48, 49] or simultaneous [50, 51, 52] multi‐slab acquisition for improved SNR efficiency.

Conducting additional Bloch simulations by introducing incremental gradient delays relative to the RF pulses, we found that gradient delays had an effect on our proposed 4D pTx SPSP pulses' performances (Figure 4), quickly degrading fat suppression with the typical ∼3 μs gradient delay as identified on a similar scanner [53]. However, our proposed 4D pulse design can readily be used to correct for gradient delays and even waveform distortions if necessary. This can be done by designing RF pulses with actual gradient waveforms measured using an MRI approach [54] or a field camera [55]. We also note that our proposed 4D pulse design can be extended for addressing high order field dynamics associated with the strong and fast switching slab‐selective gradients, for which high order field dynamics can be captured using a field camera [56].

For a proof of principle, we implemented our new pulse design using a prescribed k‐space trajectory. Part of our future work is to investigate how optimizing the k‐space trajectory (e.g., by optimizing the in‐plane gradients) would help improve the performances of our pulses in slab‐selective water‐excitation. This k‐space optimization can be done offline using an approach similar to FOCUS [57], where optimal in‐plane gradient waveforms for the application of interest are found by universal pulse design [58] with joint gradient optimization [29, 59, 60]. The optimal in‐plane gradient waveforms identified can then be used to promote online subject‐specific pulse design without increasing the computation time when compared to performing online joint gradient optimization.

For a more realistic simulation, we performed Bloch simulation at a finer discretization grid (i.e., with calibration data up‐sampled to 0.1 mm through‐plane and 0.5 mm in‐plane resolution) and integrated over voxels to simulate signal at 3 mm isotropic resolution. This was compared with our initial Bloch simulation performed at 3 mm isotropic resolution. Our results (Figure S4) showed that performing Bloch simulation at the much finer discretization followed by integrating over voxels made a little difference, leading to visually identical results. These results suggest that our proposed pulse design as implemented in the current study is robust against discretization, providing consistent performances beyond the discretized locations accounted in the pulse design. However, in general, this does not necessarily mean that good behaviors on accounted locations would guarantee identical behaviors everywhere, especially when designing more advanced pulses (e.g., with explicit SAR constraints) due in large to the increased complexity of the design problem involved. Our future work will investigate how to best design our pulses with SAR constraints while minimizing potential discretization effects.

We further characterized the spatial‐spectral response of our proposed 4D SPSP pulses and found that although targeting only a single point in the spectral domain (i.e., the water resonance at 0 Hz), our proposed 4D pulse design led to a water passband with 600 Hz bandwidth (Figure S5). Further calculation revealed that the average RMSE and CoV values inside the water passband were kept under ∼4% and 20%, respectively. We also examined the slab profiles at five frequency offsets (i.e., −450, −150, 0 and 150, 450 Hz) and found that the slab profile remained intact with *B*_0_ offsets up to ∼±150 Hz. These results suggest that our proposed 4D pulse design is robust against typical *B*_0_ offsets that may arise from inaccurate ∆*B*_0_ mapping or physiology or both.

To study how slab thickness would influence the performance of our proposed 4D SPSP pulse design, we performed two additional pulse designs targeting a narrower and a wider slab. Both pulse designs were evaluated and compared to our original pulse design targeting a slab of ∼53 mm in thickness. Our results (Figure S6) showed that for all three target slab thicknesses, our proposed pulse design led to quality slab‐selective uniform water‐excitation. These results suggest that our proposed 4D pulse design can work well for a large range of slab thicknesses, including those typically used for mesoscale fMRI studies.

A key limitation of our proposed 4D pTx SPSP pulse design is its computational intensity. This stems from addressing a comprehensive 4D problem, requiring sufficient spatiotemporal sampling and resulting in large system matrices (e.g., 5000 by 12 000 for *A*_iw_, 600 by 12 000 for *A*_ow_, 4500 by 12 000 for *A*_f_ in the pulse design shown in Figure 3). Solving such a large problem from a random initial point demands many iterations, significantly increasing computation time. To enhance efficiency, one approach could involve designing a traditional pTx spoke SPSP pulse as an initial point to reduce the iterations needed for convergence. Further investigation is warranted to assess how this strategy could improve computational efficiency for our 4D pulse design.

## 5 Conclusion

We have proposed and demonstrated a new method for designing 4D parallel transmit spatial‐spectral pulses that can be used to achieve quality uniform slab‐selective water‐excitation with an improved slab profile and without undesirable fat excitation. We have validated our new design through simulation, phantom and in vivo experiments at 7 T using the commercial Nova multi‐channel transmit RF coil. We believe our new design will have many applications including partial‐brain mesoscale functional MRI and fat‐free body imaging at UHF.

## Supporting information


**Data S1:** Supporting Information.

## Data Availability

A MATLAB implementation of our proposed 4D pulse design is made publicly available at https://github.com/longint1024/Real‐4D‐pTx.
