# Supplementary material for: Comprehensive 4D Parallel Transmission Spatial‐Spectral Pulse Design for Slab‐Selective Uniform Water‐Selective Excitation: Demonstration in the Human Brain at 7 Tesla
Source: Magn Reson Med. 2025 Nov 14;95(4):2052–62. doi: 10.1002/mrm.70183 (PMC12673673; doi:10.1002/mrm.70183)
Supplement: Supplementary file 1 — Data S1: Supporting Information. [file MRM-95-2052-s001.docx]

**Supplementary materials**

**A. Regularization parameters determination**

Here, we describe how the three regularization parameters in Eq. 1 were determined for our proposed 4D SPSP pulse design. In a nutshell, parameters λ1, λ2, and β were determined sequentially in three stages (Fig. A1). All three parameters were assigned an initial value, with the initial value of β being relatively large to ensure that the resulting peak RF amplitude would not be excessively high. In each stage, iterations were performed to adjust the parameter under consideration while keeping the other two unchanged until a prescribed stop criterion was met. In the first stage, λ1 was incremented until stopband ripples became smaller than 0.02. In the second stage, λ2 was incremented until fat residual became smaller than 0.05. In the third and last stage, β was decremented until the peak RF amplitude reached a prescribed value. The prescribed value used here was given by that of the traditional spokes pTx SPSP pulse design so that we could compare RF pulses with comparable peak amplitudes. To reduce computation burden, Eq. 1 solved in each stage was formulated at lower spatiotemporal resolution than what was desired in the final pulse design. Specifically, Eq. 1 was formulated using calibration data (i.e., dB_0_ and B1+ maps) down-sampled at 12-mm in-plane and 6-mm through-plane resolution for the region of inside-slab water, and 36-mm in-plane and 18-mm through-plane resolution for both regions of fat and out-of-slab water while assuming a dwell time of 10 $\mu s$. The computation time was ~10 minutes. Although determined using calibration data from a single volunteer, the same parameters were used to design our 4D SPSP pulses for all other volunteers.


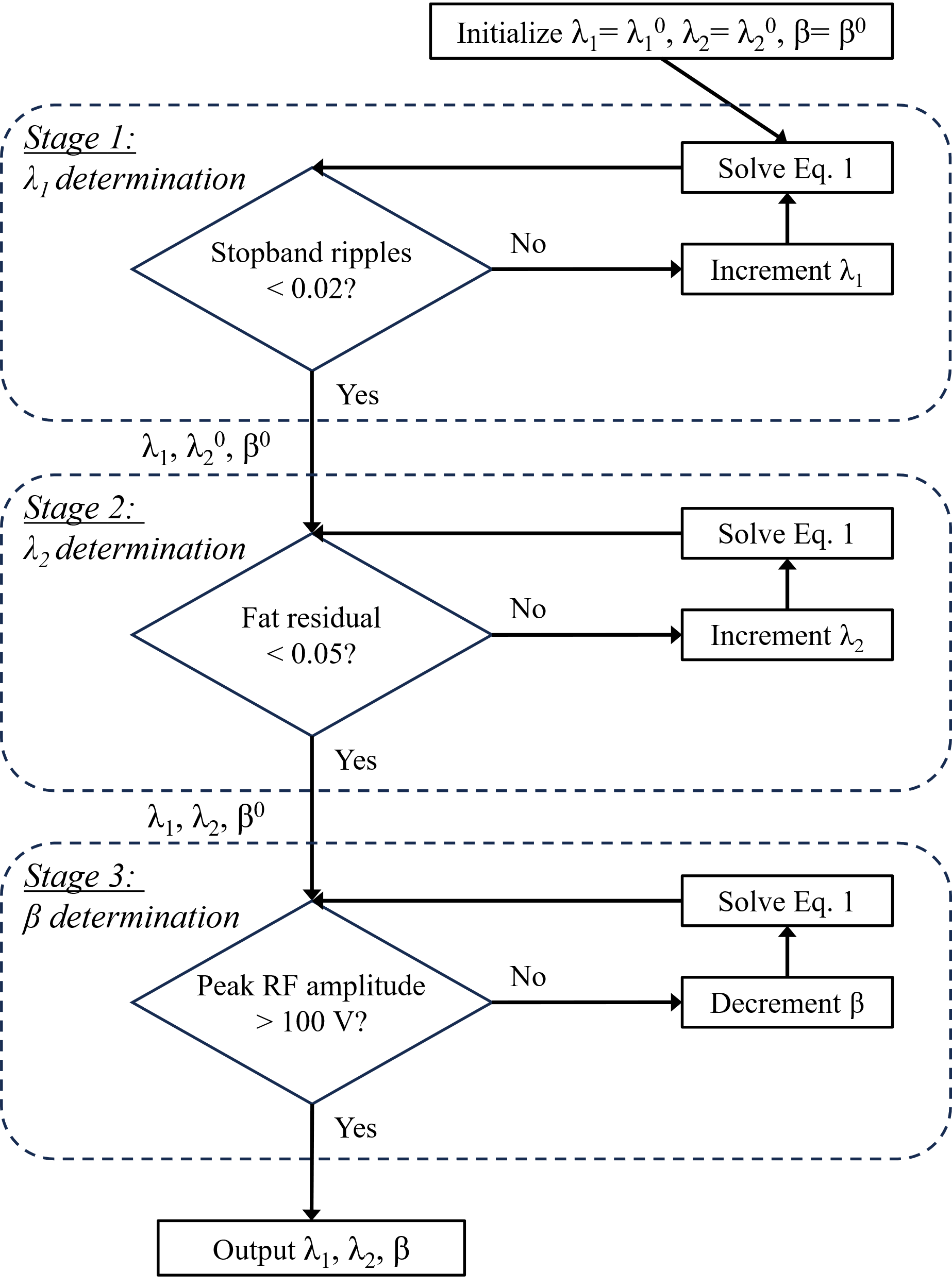


Fig. A1. Flowchart for determining the three regularization parameters in Eq. 1. Parameters were determined in three sequential stages, each stage aimed at determining one parameter while keeping the other two unchanged. Both water and fat magnetizations at equilibrium were assumed to be unity. Here initial values were λ_1_^0^=1, λ_2_^0^=1, and β^0^=5000. For faster computation, Eq. 1 here was formulated at reduced spatiotemporal resolution. The output parameters were then used to design final pulses by formulating Eq. 1 at the desired spatiotemporal resolution.

**B. Optimal TBWP comparison**

Here, we compare the optimal time bandwidth product (TBWP), that is, the highest TBWP achievable, when using monopolar vs. bipolar slab-selective gradients to design water-selective excitation RF pulses given the same inter-pulse duration and slab thickness.


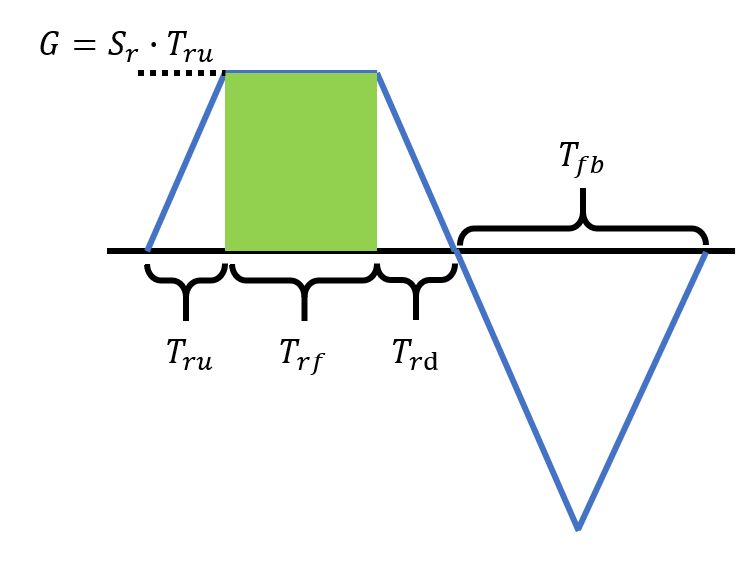


Figure A2. Schematic of one cycle of monopolar slab-selective gradients. Each cycle consists of a slab-selective gradient (including a ramp-up, a gradient plateau, and a ramp-down), followed by a fly-back. Here, $S_{r}$​ represents the slew rate, $T_{ru}$​ the ramp-up time, $G$ the gradient amplitude, $T_{rf}$​ the RF pulse duration, $T_{rd}$​ the ramp-down time, and $T_{fb}$​ the fly-back time.

Let us first derive the optimal TBWP for monopolar gradients. The total duration of one cycle of monopolar gradients (Fig. A2) determining the inter-pulse duration, $\Delta T$, is given by:

| $\Delta T=T_{ru}+T_{rf}+T_{rd}+T_{fb}$ | （A-1） |
| --- | --- |

where the definition of each term on the right-hand side is provided in the caption of Fig. A2. Furthermore, the slab-selective gradient needs to be equalized by the subsequent fly-back gradient to eliminate through-slab phases, leading to:

| $\frac{S_{r}\cdot T_{ru}^{2}}{2}+{S_{r}\cdot T_{ru}\cdot T}_{rf}+\frac{S_{r}\cdot T_{rd}^{2}}{2}=\frac{S_{r}\cdot T_{fb}^{2}}{4}$ | （A-2） |
| --- | --- |

where the left-hand side calculates the zeroth moment of the slab-selective gradient whereas the right-hand side the absolute value of that of the fly-back gradient. Combining equations (A-1) and (A-2), and assuming $T_{ru}=T_{rd}$​, we obtain:

| $T_{ru}=\frac{1}{4\Delta T}\left( \Delta T-T_{rf} \right)^{2}$ | （A-3） |
| --- | --- |

Now denoting the slab thickness as $\Delta z$, the TBWP of the RF pulse can be written as:

| ${\mathrm{TBWP}=T_{rf}\cdot\gamma G\cdot\Delta z=\gamma T}_{rf}\cdot S_{r}\cdot T_{ru}\cdot\Delta z$  $=\frac{\gamma T_{rf}S_{r}\Delta z}{4\Delta T}\left( \Delta T-T_{rf} \right)^{2}$ | （A-4） |
| --- | --- |

where $\gamma$ is the gyromagnetic ratio in units of Hz/Tesla. Equation (A-4) shows that TBWP becomes a single-variable function of $T_{rf}$ for given $\Delta T$ and $\Delta z$. Further calculation reveals that TBWP reaches its maximum value $\mathrm{TBW}P_{\mathrm{opt}}=\frac{\gamma S_{r}\cdot\Delta z\cdot\Delta T^{2}}{27}$ when $T_{rf}=\frac{\Delta T}{3}$.

Now let us derive the optimal TBWP for bipolar slab-selective gradients. Each cycle of bipolar gradients only has the slab-selective gradient with no fly-back, that is $\Delta T={2T}_{ru}+T_{rf}$. Accordingly, TBWP is given by ${TBWP=\gamma T}_{rf}\cdot S_{r}\cdot T_{ru}\cdot\Delta z=0.5\gamma T_{rf}\cdot S_{r}\cdot\Delta z(\Delta T-T_{rf})$. The optimal TBWP is $\mathrm{TBW}P_{\mathrm{opt}}=\frac{\gamma S_{r}\cdot\Delta z\cdot\Delta T^{2}}{8}$ when $T_{rf}=\frac{\Delta T}{2}$.

Our calculations of optimal TBWP show that using bipolar slab-selective gradients will increase the optimal TBWP by more than threefold when keeping the inter-pulse duration and slab thickness constant. For water selective excitation, the inter-pulse duration should be short enough to allow effective separation of water and fat resonances and can be given by $\Delta T=\frac{1}{2\delta\gamma B_{0}}$ where $\delta$ is the chemical shift determined water-fat separation and $B_{0}$ the main magnetic field strength. This leads to $\mathrm{TBW}P_{\mathrm{opt}}=\frac{S_{r}\cdot\Delta z}{32\gamma\delta^{2}{\cdot B}_{0}^{2}}$ for bipolar and $\mathrm{TBW}P_{\mathrm{opt}}=\frac{S_{r}\cdot\Delta z}{108\gamma\delta^{2}{\cdot B}_{0}^{2}}$ for monopolar gradients. Now consider a pulse design (Fig. S1) with $S_{r}=160$ Tesla/m/s (lower than the maximum slew rate to be conservative), $\Delta z=0.04$ m, $\gamma=$42.58 MHz/Tesla, $\delta=3.35$ ppm, and $B_{0}=7$ Tesla, the optimum TBWP is calculated to be ~8.54 for bipolar, but only ~2.53 for monopolar slab-selective gradients.

**Supplementary Figures**


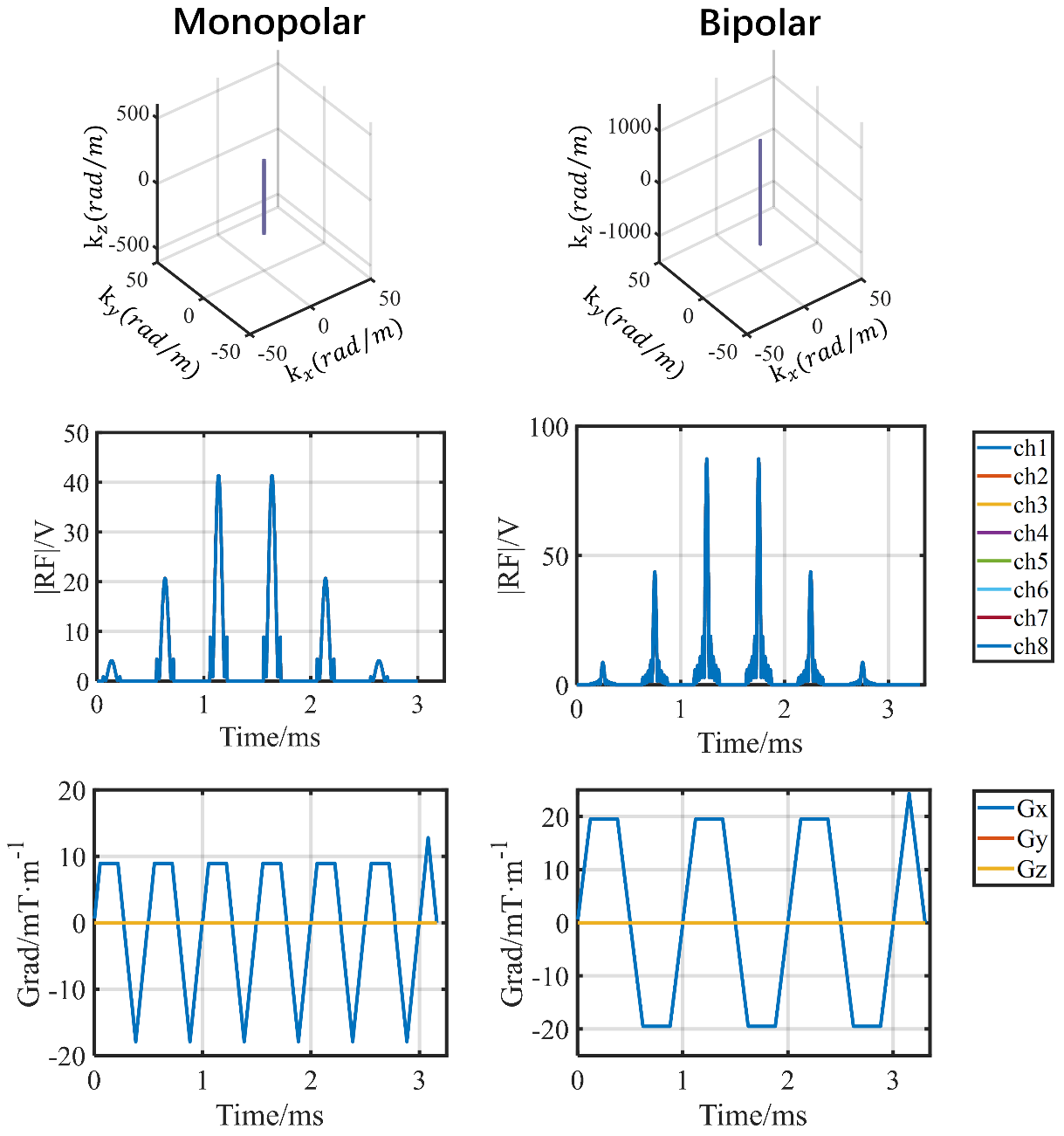


**Fig. S1.** Example binomial pulse designs with monopolar vs. bipolar slab-selective gradients. For each, displayed are k-space trajectories (top), RF amplitudes (middle), and corresponding gradient waveforms (bottom). Both composite pulses were designed with the same nominal slab thickness of 48 mm using the same binomial coefficients of 1-5-10-10-5-1 for water-excitation. To keep the same inter-pulse duration, shorter sub-pulses had to be used in forming the composite pulse with monopolar gradients due to the presence of flyback gradients. This resulted in a reduced TBWP (2.8 vs. 9.8 for the composite pulse with bipolar gradients) when the slab-selective gradients applied in both composite pulse designs were designed with their respective optimal TBWP.
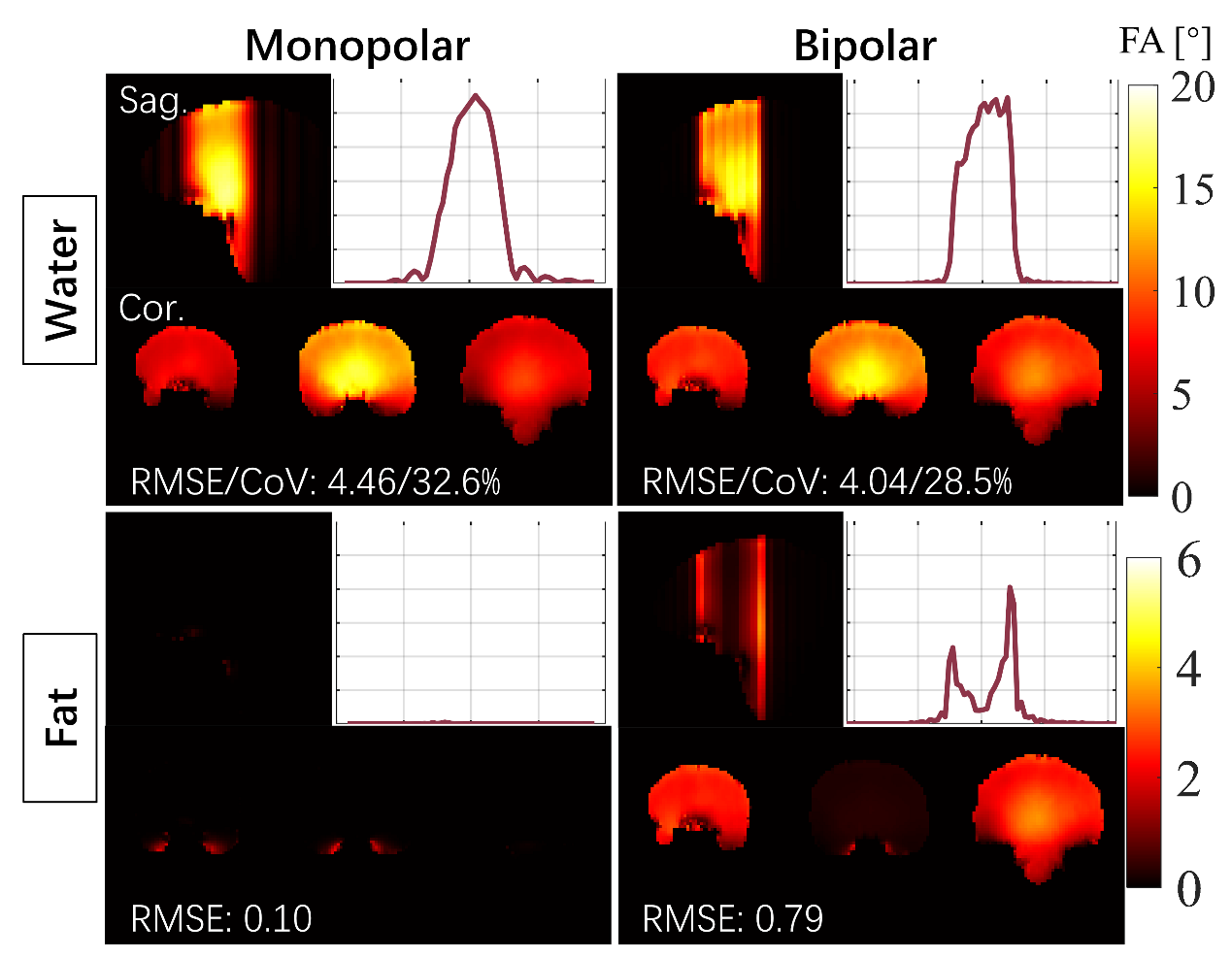


**Fig. S2.** Comparing the slab-selective water-excitation performances of the two example binomial pulse designs as shown in Fig. S1. Shown are Bloch simulated flip angle maps in the middle sagittal slice (alongside corresponding slab profiles) and three representative coronal slices for water (top) and fat (bottom) resonances, assuming a human scan using the commercial Nova 8Tx32Rx head RF coil operating in its CP mode mimicking a single-channel transmit setup. For each pulse design scenario, RMSE values were computed to evaluate performances for water excitation (calculated with respect to the Ernst angle of ~13°) and fat suppression (calculated with respect to 0°). CoV values were also computed to quantify flip angle homogeneity for within-slab water excitation. Note how the use of monopolar slab-selective gradients appeared to degrade the slab profile, despite that it was effective at minimizing the fat odd lobes.


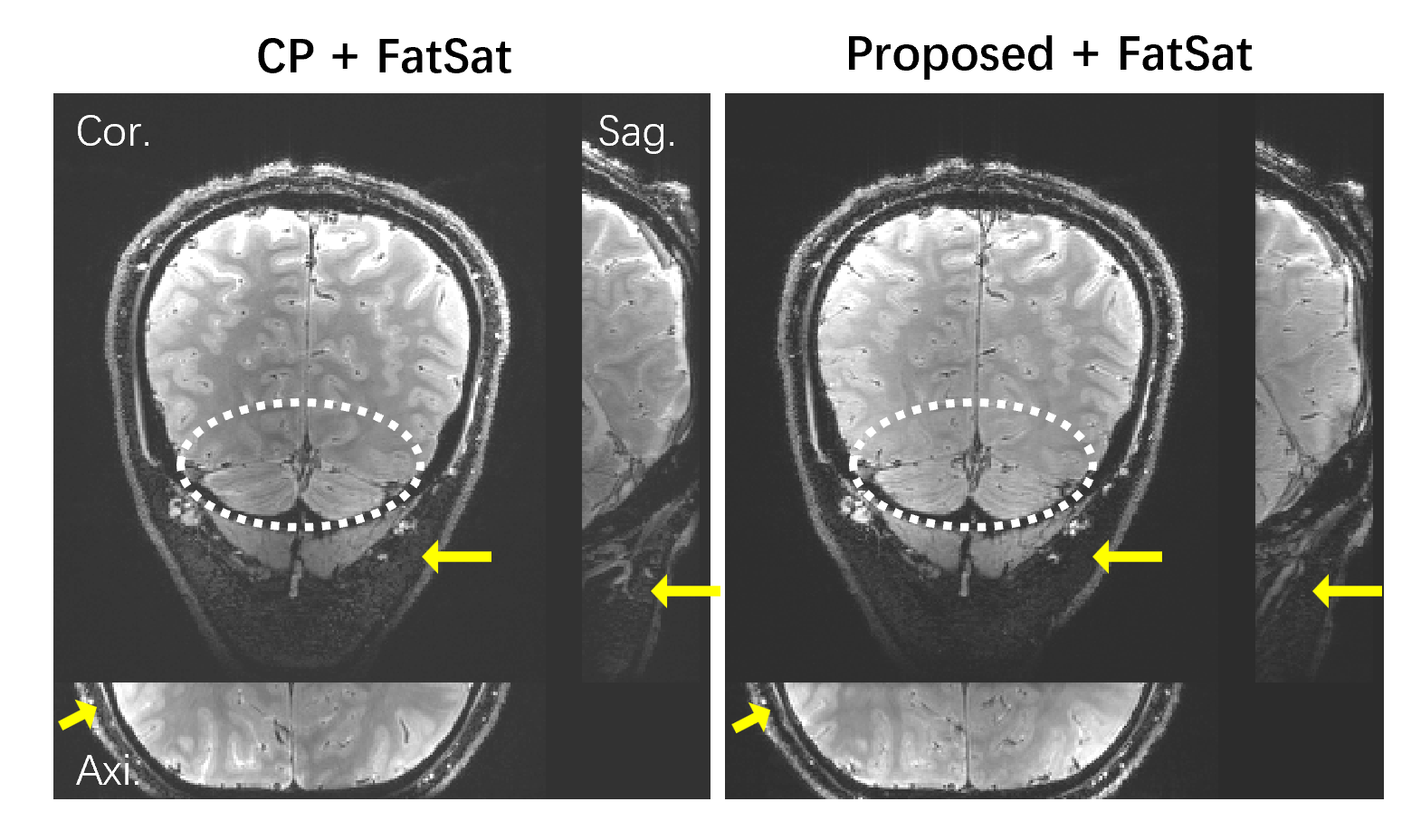


**Fig. S3.** Improving fat suppression performances by using additional fat saturation (FS). Shown are single-slab 3D-GRE images in three orthogonal views acquired using our proposed pulse design coupled with vendor-provided FS in the CP mode (Proposed + FS), in comparison to regular slab-selective excitation in the CP mode with the same FS (CP + FS). GRE images were obtained using same parameters as in Fig. 6. Note how our proposed pulse design outperformed the traditional CP-mode based approach, producing better fat-suppression (as indicated by arrows) while restoring signal in lower brain (as indicated by ovals).


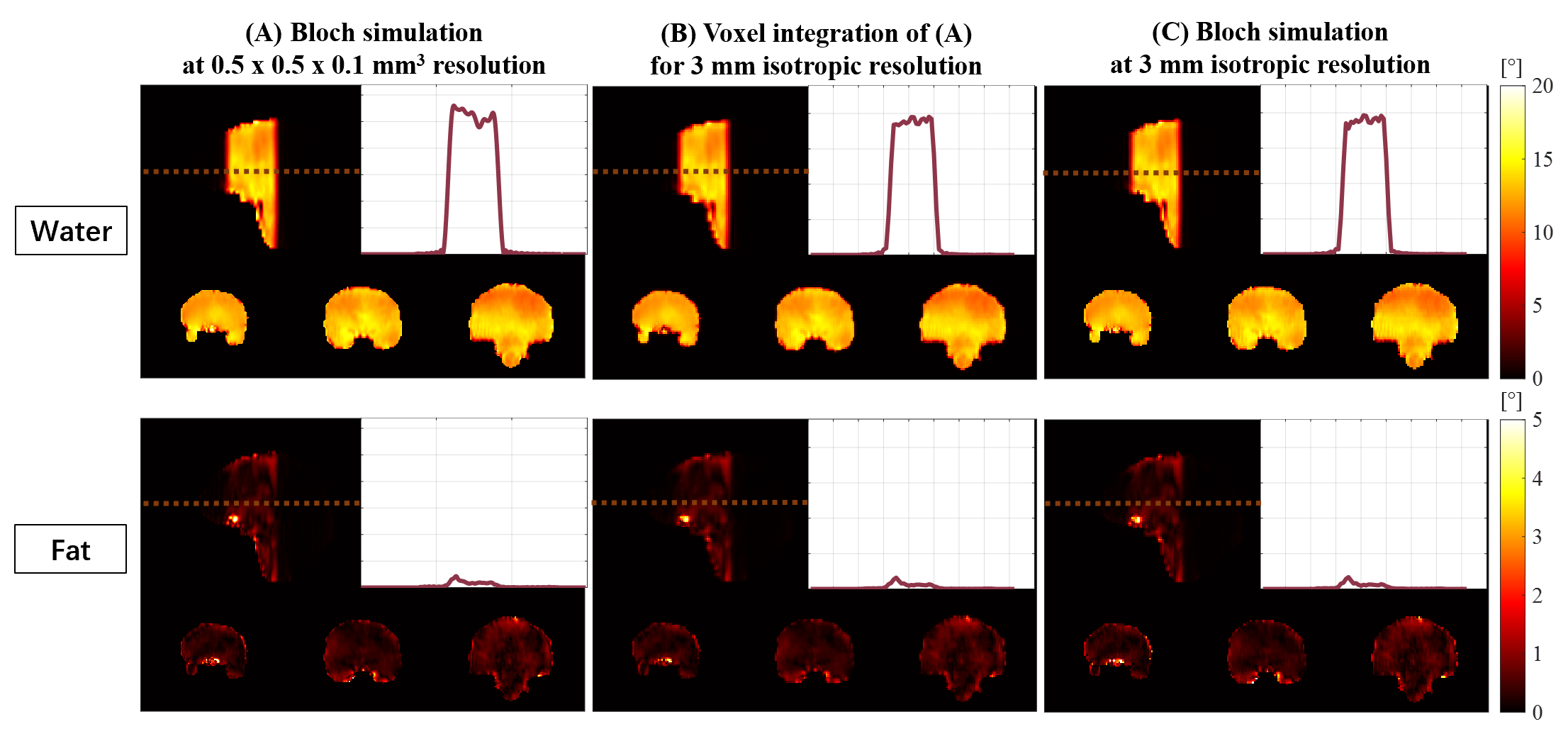


**Fig. S4.** Comparing the performances of our proposed pulses when designed with calibration data resampled at coarse, moderate and fine resolution. Shown are Bloch simulated flip angle maps in the middle sagittal slice (alongside corresponding slab profiles) and three representative coronal slices for water (top) and fat (bottom) resonances for pulses designed based on the calibration data obtained from the same volunteer. The moderate calibration data were the one used in the current study. The coarser calibration data were obtained by further under-sampling the moderate one by a factor of 2, i.e., resampled at 12 mm in-plane and 6 mm through-plane resolution for inside-slab water, and 36 mm in-plane and 18 mm through-plane resolution for regions of fat and out-of-slab water. The finer calibration data were obtained by up-sampling the moderate one by a factor of 2, i.e., resampled at 3 mm in-plane and 1.5 mm through-plane resolution for inside-slab water, and 9 mm in-plane and 4.5 mm through-plane resolution for regions of fat and out-of-slab water. All Bloch simulations were performed using the same 3 mm calibration data. Note that using coarser calibration data degraded the performances of our pulses (especially for fat suppression) and using finer calibration data marginally improved our pulses’ performances.


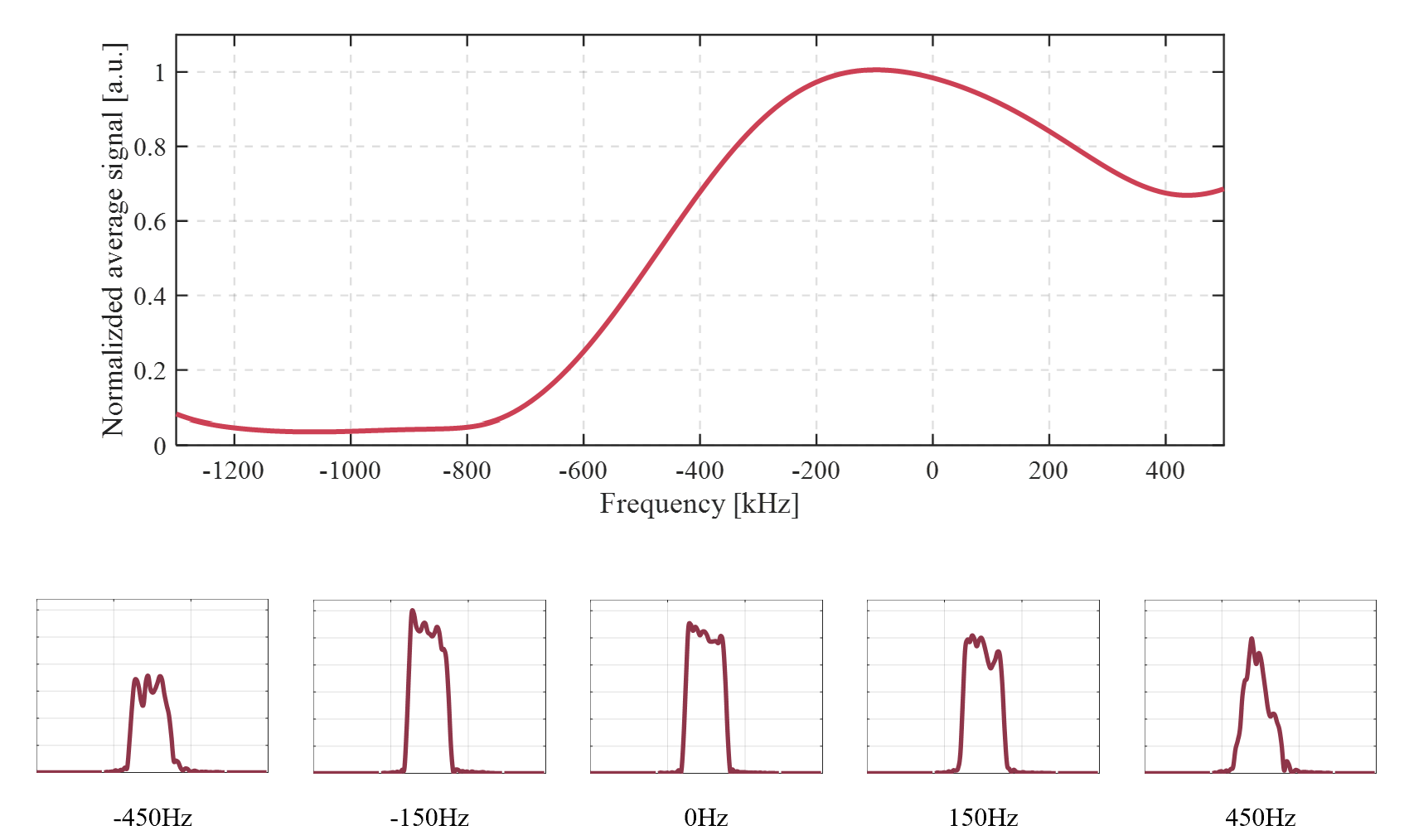


**Fig. S5.** Examining the spatial spectral response of our proposed 4D pTx SPSP pulses. Although designed to excite only the water resonance at 0 Hz, the resulting water passband exhibited a bandwidth of ~600 Hz (top). Within this passband, the average RMSE and CoV remained below ~4 and 20%, respectively. Slab profiles acquired at frequency offsets of −450, −150, 0, 150, and 450 Hz (bottom) showed minimal degradation up to ~±150 Hz, indicating robustness of the proposed 4D design to typical B₀ offsets arising from ∆B₀ mapping inaccuracies or physiological variations.


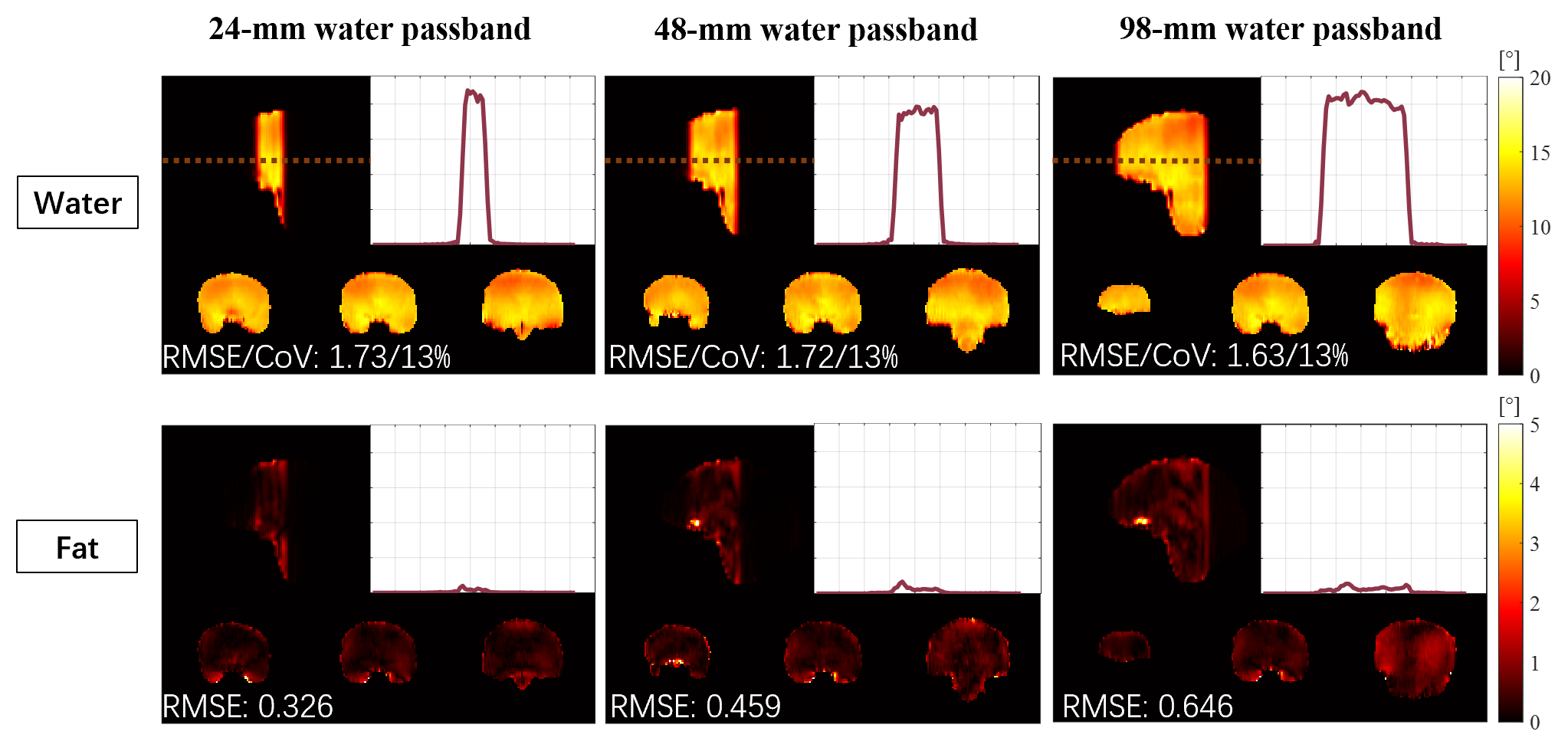


**Fig. S6.** Evaluating the effect of slab thickness in our proposed 4D pTx SPSP pulse design. Bloch simulations and excitation slab profiles are shown for 24-mm and 98-mm water passbands (top row) along with corresponding fat-frequency flip angle maps (bottom row). Across both slab thicknesses, the pulses achieved uniform slab-selective water excitation with low RMSE/CoV values (~1.6-1.7/13%) and good fat suppression, demonstrating robustness over a wide range of slab sizes, including those relevant for mesoscale fMRI.
